# Novel Strategies to Reduce Pulmonary Hypertension in Infants With Bronchopulmonary Dysplasia

**DOI:** 10.3389/fped.2020.00201

**Published:** 2020-05-08

**Authors:** Ahmed El-Saie, Binoy Shivanna

**Affiliations:** Department of Pediatrics, Section of Neonatology, Baylor College of Medicine, Texas Children's Hospital, Houston, TX, United States

**Keywords:** bronchopulmonary dysplasia, pulmonary hypertension, pathogenesis, diagnosis, novel therapeutic strategies, preclinical studies

## Abstract

Bronchopulmonary dysplasia (BPD) is a developmental lung disorder of preterm infants primarily caused by the failure of host defense mechanisms to prevent tissue injury and facilitate repair. This disorder is the most common complication of premature birth, and its incidence remains unchanged over the past few decades. Additionally, BPD increases long-term cardiopulmonary and neurodevelopmental morbidities of preterm infants. Pulmonary hypertension (PH) is a common morbidity of BPD. Importantly, the presence of PH increases both the short- and long-term morbidities and mortality in BPD infants. Further, there are no curative therapies for this complex disease. Besides providing an overview of the pathogenesis and diagnosis of PH associated with BPD, we have attempted to comprehensively review and summarize the current literature on the interventions to prevent and/or mitigate BPD and PH in preclinical studies. Our goal was to provide insight into the therapies that have a high translational potential to meaningfully manage BPD patients with PH.

## Introduction

Bronchopulmonary dysplasia (BPD), the most common infantile chronic lung disease in the US, is still one of the most challenging complications of preterm infants. The histopathological hallmarks of this disease are alveolar simplification and dysmorphic pulmonary vascularization (1, 2). The definition of BPD continues to be very challenging and evolving as a result of the ongoing advances in the respiratory management of preterm infants (3–10). With increasing survival of extremely preterm infants, the incidence of this disease remains high (11–14). Further, BPD poses a major clinical, social, and economic problem (15, 16). Infants with this disease have long-term pulmonary, cardiovascular, and neurodevelopmental abnormalities (17–21). The hospitalization cost of BPD infants is twice that of non-BPD infants in the 1st year of life (16). Pulmonary hypertension (PH) is a common morbidity of BPD. The pooled prevalence rates of PH in mild, moderate, and severe BPD are 6, 12, and 39%, respectively (22). Importantly, the presence of PH increases both the short- and long-term morbidities and mortality in BPD infants (22–26). In this review, we provide a summary of the literature on the pathogenesis of BPD and PH and the potential strategies to prevent and manage this disease complex.

## Methods for Literature Search

We searched PubMed and MEDLINE databases through the time period 1990 to present. The following mesh terms without any language restrictions were included in our search: “Bronchopulmonary Dysplasia” AND “Pulmonary Hypertension” OR “Bronchopulmonary Dysplasia” AND “Pulmonary Hypertension” AND “Pathogenesis” OR “Bronchopulmonary Dysplasia” AND “Pulmonary Hypertension” AND “Diagnosis” OR “Bronchopulmonary Dysplasia” AND “Pulmonary Hypertension” AND “Treatment OR Therapy OR Management,” OR “Bronchopulmonary Dysplasia” AND “Pulmonary Hypertension” AND “Novel Therapeutic Strategies” OR (“Bronchopulmonary Dysplasia” AND “Pulmonary Hypertension” OR Lung Injury”) AND (“Preclinical Studies” OR “Animals”). The two authors (AE-S and BS) independently evaluated the titles and abstracts of the studies for eligibility for inclusion in this review. We obtained the full-text version for assessment and resolved any differences by discussion.

## Pathogenesis

The pathophysiology of PH in BPD is complex and is characterized by decreased pulmonary blood vessel density, endothelial cell dysfunction, and increased remodeling and altered vasoreactivity of resistance pulmonary arteries (blood vessels with a diameter of <150 μm) (27). The pathogenesis of this disease complex is multifactorial, and the pathogenic factors can be prenatal, natal, or postnatal in origin. The majority of these risk factors are common for both BPD and BPD with PH (25). Here, we attempt to briefly summarize the factors that predominantly predispose infants to develop BPD and PH. Placental abnormalities, especially those associated with hypo- or under-perfusion, increase the risk of BPD and PH (28, 29). Placental abnormalities are associated with decreased levels of proangiogenic and increased levels of anti-angiogenic molecules (30–32), which can inhibit angiogenesis and cause endothelial cell dysfunction at birth. Not surprisingly, infants born to mothers with such placental vascular anomalies are at high risk of BPD and PH because of the well-established association between the structural and functional abnormalities of the lung vasculature and the development of BPD (33). Fetal growth restriction, regardless of its severity, is a significant antenatal risk factor for the development of PH in BPD infants, whereas maternal hypertensive disorders without fetal growth restriction do not increase the risk of BPD and PH (34). Other prenatal risk factors for BPD and PH include genetic abnormalities, oligohydramnios, and maternal diabetes (35–42). Natal risk factors include preterm birth, low birthweight, and low 5-min Apgar scores (35, 39). Postnatal risk factors for this disease complex include oxidative stress, mechanical ventilation, infection, and hemodynamic overcirculation (42, 43).

## Diagnosis and Evaluation

Cardiac catheterization remains the gold standard for the evaluation and diagnosis of PH (25). Catheterization is particularly helpful in detecting response to vasodilating agents and oxygen therapy (44). It is also helpful in characterizing pathologies such as pulmonary stenosis and aortopulmonary collaterals, in addition to left ventricular dysfunction (25). However, catheterization is invasive, requires general anesthesia, and carries several risks, especially in small infants. Therefore, the procedure should be considered wisely in infants with BPD and PH (44).

Echocardiography is the most widely used tool for the evaluation of pulmonary artery pressure because it is non-invasive and easily accessible. However, the optimal time to screen BPD infants for PH by echocardiography is uncertain. Considerable heterogeneity in the timing to screen for PH exists in neonatal intensive care units across the world. The consensus is to screen infants with moderate and severe BPD at 36 weeks postmenstrual age or infants requiring significant respiratory support with recurrent hypoxemic episodes at any age (45, 46). To obtain meaningful information, the echocardiography should evaluate and quantify the structural and pulmonary venous abnormalities; shunts; ventricular size, hypertrophy, and function in both systole and diastole; interventricular septal position in systole and diastole; and tricuspid and pulmonary regurgitation jet velocities (TRJV). Further, the systemic blood pressure values should be documented during the echocardiography screen (45). The TRJV is commonly used to estimate PH by echocardiography using a modified Bernoulli equation (47). A limitation of the TRJV measurement is that it is only detected in 61% of infants with BPD and PH, and its absence does not exclude PH (44, 48). Evaluation of other parameters like interventricular septum flattening and right ventricular hypertrophy or dilatation can be subjective and inaccurate. In patients with PH, it is recommended to evaluate the right and left ventricular function. The left ventricular eccentricity index measurements can be used to identify BPD infants suffering from PH. As the pulmonary pressure rises, it causes right ventricular overload and septal distortion. The left ventricular eccentricity index quantifies the septal distortion at end-systole and end-diastole and is reported to be significantly higher in patients with PH and BPD than those with BPD alone (49). Right ventricle (RV) systolic function can also be assessed by quantifying the tricuspid annular plane systolic excursion (TAPSE) (50). The TAPSE correlates directly with the gestational age (GA) and increases with advancing GA (51). In BPD infants with PH, the TAPSE is significantly decreased (52, 53). Pulmonary artery acceleration time (PAAT) is a reliable measure of RV afterload and, therefore, the pulmonary arterial pressure in infants and children (54). The PAAT and PAAT/RV ejection time are decreased in BPD infants with PH (55, 56). For detection of certain pathologies that are not identified by conventional methods, tissue Doppler and speckle tracking echocardiography have been suggested (57). Specifically, speckle tracking echocardiography is used in infants and children with PH to identify RV longitudinal strain, a parameter of RV systolic function. The strain measures the degree of myocardial deformation over time and is directly proportional to the myocardial contractility and ventricular systolic function (58). RV longitudinal strain is decreased in BPD infants with PH (59). It is also important to interrogate the pulmonary veins by Doppler echocardiography in BPD infants who have PH because pulmonary vein stenosis (PVS) is increasingly recognized in these infants (60, 61). Further, PVS develops over time, occurs more frequently in infants with necrotizing enterocolitis, and increases the mortality rate (62, 63).

Magnetic resonance imaging (MRI) can be a useful non-invasive imaging modality to characterize BPD and its associated complications, including PH (64). With advancements in MRI technology over the years, the scanning time and the need for sedation during imaging have been substantially decreased, making it a promising imaging modality for infants with BPD. While MRI has been used to evaluate PH in adults, its use in neonates and infants remains under investigation. Cardiac MRI is a three-dimensional imaging study that accurately quantifies the architectural details of the cardiac chambers, including their respective volumes, mass, and transvalvular flow (65). Recently, Critser et al. investigated whether cardiac MRI would predict BPD severity and identify the infants with BPD who required therapy for PH (26). The investigators used MRI to measure several parameters of cardiac structure and function, including the main pulmonary artery-to-aorta diameter (PA/AO) ratio, left ventricular eccentricity index (MR-EI), left and right ventricle end-systolic volumes, right ventricular end-diastolic volume, ejection fraction, and the cardiac index. Further, they used measurements obtained by clinically indicated echocardiography studies and compared the MRI and echocardiographic measurements with the following outcomes: BPD severity, length of hospital stay (LOS), duration of respiratory support, the need for respiratory support at discharge, and the need for PH therapy. The investigators observed that increased PA/AO ratio, MR-EI, and echocardiography-EI correlated with the predetermined clinical outcomes; however, there was no correlation between pulmonary arterial blood flow and the BPD outcomes. After controlling for the confounding variables, MR-EI predicted the LOS and duration of respiratory support, whereas the PA/AO ratio and MR-EI predicted the need for PH therapy both during hospitalization and at discharge. Despite the study limitations, the findings suggest that cardiac MRI may be useful to manage BPD infants with PH.

Serum brain natriuretic peptide (BNP) and its prohormone N-terminal-proBNP are released by the cardiomyocytes in response to neurohormonal factors (66) and are used for the diagnosis and monitoring of infants with PH in the context of BPD (67–69). Asymmetric dimethylarginine, endostatin, and angiopoietin are the other molecules that are abnormally expressed in BPD patients with PH (37, 70).

Importantly, infants with BPD and PH should be evaluated for comorbidities that could initiate and potentiate the pulmonary vascular disease. These morbidities include aspiration, gastroesophageal reflux disease, structural airway disease (vocal cord paralysis, subglottic stenosis, tracheomalacia, bronchomalacia, and airway hyperreactivity), PVS, left ventricle (LV) dysfunction, and aortopulmonary collaterals (45).

## Management

Multidisciplinary care provided by a team of neonatologists, cardiologists, PH specialists, nursing staff, respiratory therapists, pharmacists, dieticians, occupational, and developmental specialists should be the norm for the comprehensive management of BPD infants with PH.

General management principles include strategies to avoid intermittent or chronic increases in the pulmonary vascular resistance PVR), treat comorbidities (45), provide optimal nutrition to allow the lungs to repair and recover from the chronic injury (7), and promote developmental support care to improve the neurodevelopmental outcomes (71). Maintaining oxygen saturation (SpO_2_) between 92 and 95% (72), avoiding hypercapnia, and preventing lung over- or under-inflation are the general approaches to decrease the PVR (73).

Data related to the dose, route of administration, efficacy, and safety of specific pulmonary vasodilators in BPD patients with PH are limited and continue to evolve. Increased pulmonary vascular pressures and RV dysfunction that persist after treating the comorbidities and excluding LV dysfunction and PVS are indications to initiate specific pulmonary vasodilator therapy in BPD infants (45). Inhaled nitric oxide (iNO) is commonly used to treat PH associated with BPD; however, the only FDA-approved indication of this medication in neonates is to treat infants >34 weeks' gestation with hypoxic respiratory failure secondary to PH (25, 74). Therapy with iNO selectively vasodilates the pulmonary vasculature, decreases PVR, and improves oxygenation in BPD infants who have PH (75, 76). There is limited data regarding the use of phosphodiesterase inhibitors (sildenafil and milrinone), endothelin receptor antagonist (bosentan), and prostacyclins and their analogs for the management of PH in BPD infants (25, 45).

## Novel Experimental Therapies

This section includes data from preclinical studies that have modeled human BPD with PH, quantified the PH phenotype, and demonstrated the effects of the intervention on lung development and PH (Table 1).

**Table 1:** Overview of the novel therapeutic interventions in experimental bronchopulmonary dysplasia and pulmonary hypertension.

**Therapeutic targets**	**Species**	**Exposure**	**Phenotype(s)**	**References**
Connective tissue growth factor (CTGF)	Mice and rats	Hyperoxia	Increased CTGF signaling potentiates hyperoxia-induced alveolar and pulmonary vascular simplification and PH, whereas the converse is true with decreased CTGF signaling.	(77–80)
Soluble fms-like tyrosine kinase 1 (sFlt-1)	Rats	Intramniotic injections of sFlt-1 or endotoxin	Anti-sFlt-1 monoclonal antibody improves alveolarization and lung vascularization and decreases PH in antenatal models of experimental BPD.	(81)
Exosomes	Mice	Hyperoxia	Human mesenchymal stromal cell exosomes attenuate hyperoxia-induced experimental BPD, PH, and lung dysfunction.	(82, 83)
Interleukin-1 receptor antagonist (IL-1RA)	Mice	Hyperoxia	Inhibition of IL-1 signaling abrogates hyperoxia-induced disrupted lung development, pulmonary vascular resistance, and cardiac fibrosis.	(84)
Microbiome	Rats and mice	Postnatal growth restriction (PNGR) and hyperoxia	PNGR alone causes intestinal dysbiosis and PH without BPD, but hyperoxia + PNGR potentiates PH and induces BPD. Probiotics mitigate PH in PNGR mice. The alveolarization is severely disrupted in non-germ-free mice, but the PH phenotype is comparable between non-germ-free and germ-free mice.	(85–87)
Stem cells and their conditioned media	Rats, mice, and human infants	Hyperoxia	Both stem cells and their conditioned media rescue rodent lungs from hyperoxia-induced inflammation, fibrosis, dysfunction, and stunted development; however, only the stem cells rescue the PH phenotype. Infants tolerate stem cell therapy.	(88–92)
Stromal-derived factor-1 (SDF-1)	Rats	Hyperoxia	SDF-1 attenuates hyperoxia-induced alveolar and pulmonary vascular simplification and PH.	(93)

### Anti-connective Tissue Growth Factor Antibody

Connective tissue growth factor (CTGF) is a transforming growth factor beta target gene that regulates cell proliferation, adhesion and migration, extracellular matrix deposition, angiogenesis, and development of the skeletal system (94–97). Aberrant expression of CTGF is associated with tissue fibrosis and remodeling (98, 99). Further, the expression of this growth factor is increased in animal models and infants with BPD (77, 100, 101). Mechanistic preclinical studies have consistently demonstrated that CTGF disrupts postnatal lung development and potentiates experimental BPD with PH (78–80). Conversely, the administration of a neutralizing CTGF antibody to rats mitigates hyperoxia-induced alveolar and pulmonary vascular simplification as well as PH (77).

### Anti-soluble fms-Like Tyrosine Kinase 1 Antibody

Soluble fms-like tyrosine kinase 1 (sFlt-1) is a soluble vascular endothelial growth factor (VEGF) receptor-1 that exerts anti-angiogenic effects by acting as an endogenous VEGF inhibitor (102–104). Elevated levels of this receptor increase the risk and severity of BPD in both animals (81) and humans (105). Using a rigorous approach, Wallace et al. (81) demonstrated that both prophylactic and rescue therapies with an anti-sFlt-1 monoclonal antibody in rats are effective in increasing alveolarization and lung vascularization and decreasing right ventricular hypertrophy in two prenatal models of BPD. Importantly, these investigators modeled preeclampsia and chorioamnionitis, the two major antenatal determinants of BPD in infants. Further, their molecule of interest was targeted to a pathway that has been well-established to play a role in BPD pathogenesis. Therefore, targeting sFlt-1 has a high translational potential in infants with BPD and PH who have increased expression of sFlt-1.

### Exosomes

Exosome, a subtype of extracellular vesicle, is a plasma membrane-enclosed signaling vector secreted *via* the endosomal pathway. Their enriched bioactive cargo, including small non-coding RNAs, free fatty acids, surface antigens, and protein, allows them to be one of the most effective mediators of cell signaling (106–108). The number and miRNA signatures of these exosomes are altered in BPD infants (109), indicating that these vesicles may play a pathogenic role and can be targeted to develop therapies. Two recent preclinical studies strongly suggest that exosomes can be an effective therapy for BPD infants with PH. In a murine model of hyperoxia-induced BPD and PH, Willis and colleagues (82) elegantly demonstrated that exosomes, purified from the mesenchymal stromal cells of both human bone marrow and umbilical cord Wharton's jelly, attenuated pulmonary vascular remodeling, PH, and lung fibrosis and improved lung development and function in mice exposed to hyperoxia. Similarly, Chaubey et al. (83) demonstrated that umbilical cord-derived exosomes ameliorate hyperoxia-induced lung inflammation, alveolar simplification, and PH in neonatal mice. Although recent advances in processes such as isolation, purification, and characterization of the exosomes have increased our understanding of these vesicles in health and disease, the advances are still at an infancy stage, and there is a need for improvement and standardization of these processes before we can definitely conclude on the detrimental and beneficial effects of exosomal therapy (110).

### Interleukin-1 Receptor Antagonist

Interleukin-1 (IL-1) is a cytokine that is implicated in the patho genesis of many acute and chronic inflammatory diseases. Not surprisingly, elevated levels of this cytokine are associated with increased BPD incidence in infants (111–113). It is also one of the few cytokines that have been directly implicated in the pathogenesis of experimental BPD (114–118). Bui et al. (84) recently demonstrated for the first time that IL-1 receptor antagonist (IL-1Ra) decreases both the short- and long-term adverse effects of neonatal hyperoxia on pulmonary vasculature in mice. Using elegant and robust methods, they showed that IL-1Ra improves pulmonary vascular density and alveolarization and decreases pulmonary vascular resistance and cardiac fibrosis. These observations indicate that IL-1Ra attenuates murine BPD and PH. The antagonist was also recently shown to be safe and effective in adult patients with PH and right ventricular failure (119), emphasizing the translational potential of this compound for BPD infants with PH.

### Microbiome

Dysbiosis, or a disruption in the balance between the structure of complex microbial communities on or inside the body, plays a major role in the pathogenesis of several inflammatory diseases (120). We now know that the human respiratory tract microbial colonization begins *in utero* (121, 122) or shortly after birth (123, 124). Chorioamnionitis, antibiotic exposure, mode of delivery, method of feeding, and bowel colonization can decrease bacterial diversity and increase pathogenic microbial colonization in the lungs (125), increasing the risk of lung inflammation and BPD. Two recent preclinical studies highlight the role of microbiota in the pathogenesis of BPD and PH. Postnatal growth restriction (PNGR) causes PH without disrupted lung development in neonatal rats (85). However, when these growth-restricted rats are exposed to hyperoxia, they also develop alveolar simplification and have a severe PH phenotype. Further, Wedgwood et al. showed that PNGR, but not hyperoxia, independently alters intestinal microbiota in the same model, and mitigation of this intestinal dysbiosis with a probiotic alleviates the PH in neonatal PNGR mice exposed to normoxia or hyperoxia (86). To elucidate the pathogenic role of microbiota in BPD, Dolma et al. (87) exposed germ-free (GF) and non-germ-free (NGF) mice to 21% FiO_2_ (normoxia) or 85% FiO_2_ (hyperoxia) for up to postnatal day 14, which is a well-established murine model of experimental BPD. At baseline, lung development was comparable between GF and NGF mice. However, hyperoxia-induced interruption in lung development was significantly worse in NGF than in GF mice, indicating that pathogenic bacteria can worsen experimental lung injury. Interestingly, the severity of PH was similar in hyperoxia-exposed GF and NGF mice, suggesting that microbiota may not play a major role in the pathogenesis of hyperoxia-induced PH. The above two studies indicate the need for further robust studies to identify the select population of BPD infants with PH who may benefit from therapies targeting the microbiota.

### Stem Cells and Their Conditioned Media

There has been an increased focus on stem cell biology for discovering therapies for BPD and PH because the very biological effects of stem cells such as self-renewal and differentiation into specialized cell types (126) make them ideal candidates to promote organ development and repair. Further, stem cell biologists have focused mainly on and used mesenchymal stem cells (MSCs) in experimental lung injury due to reasons such as the feasibility of isolation and the pleiotropic effects of these cells (127). Other types of stem cells used for intervention in experimental BPD include endothelial colony forming cells, endothelial progenitor cells, bone marrow-derived angiogenic cells, mononuclear and cord blood CD34^+^ cells, human amniotic fluid stem and epithelial cells, bone marrow derived ckit^+^ cells, and human-induced pluripotent stem cells (128). The major source of MSCs used in experimental models of BPD has been the bone marrow, while the predominant route of administration of the cells in these models has been the intratracheal route (88). Systematic analyses of 25 preclinical studies on MSC therapy in BPD (88) indicated that MSCs improved lung alveolarization regardless of the source and route of administration. The cell therapy also decreased lung inflammation, apoptosis, fibrosis, pulmonary vascular remodeling, and pulmonary hypertension and improved lung function (compliance, elastance, total lung capacity, and inspiratory capacity). Therapy with the conditioned media from these MSCs was also effective in improving alveolarization and lung vascularization and in reducing lung inflammation and fibrosis. Interestingly, the systematic review indicated that, unlike MSCs, their conditioned media do not improve the PH (88). A similar conclusion was drawn from another systematic review of preclinical studies that used MSC-conditioned media for various lung diseases (129). Only two studies (89, 90) on experimental BPD met the criteria for inclusion in these analyses. The anti-inflammatory effects of the conditioned media were comparable to MSCs. Further, the conditioned media also improved alveolarization. Recently, Moreira et al. for the first time, demonstrated the safety and efficacy of intranasal human umbilical cord-derived MSCs in experimental BPD (91). The first trial of stem cell therapy for BPD infants was conducted by Lim et al. from Australia. These investigators demonstrated that allogenic amniotic cells could safely be administered intravenously to preterm infants with BPD (*n* = 6) (92). A follow-up of these infants (*n* = 5) for 2 years indicated that there were no long-term adverse consequences of amniotic cell therapy (130). Similarly, Powell et al. showed that MSCs from human umbilical cord blood were well-tolerated without any adverse effects in extremely low-birth-weight infants (*n* = 12) when administered by the intratracheal route (131). Based on all these studies and systematic reviews, MSCs or their conditioned media are excellent therapeutic candidates for BPD infants.

### Stromal-Derived Factor-1

Stromal-derived factor-1 (SDF-1) is a chemokine that signals through the receptors, chemokine receptors 4 and 7, and modulates cell migration, proliferation, and angiogenesis, the biological processes that are crucial for organ development and repair (132–137). In neonatal rats, Guerra et al. (93) recently showed that SDF-1 is expressed in both lung epithelial and endothelial cells, and hyperoxia decreases the expression of this chemokine. Further, using a non-viral form of gene therapy, they elegantly demonstrated that SDF-1 delivery to the lungs improves angiogenesis and alveolarization, decreases pulmonary vascular remodeling and inflammation, and attenuates experimental BPD and PH induced by hyperoxia. Importantly, this non-viral form of gene therapy is proven to be safe and effective in humans (138) and, therefore, has a high translational potential to target therapies to the diseased lung while avoiding off-target effects.

## Conclusions

BPD with PH remains a significant short- and long-term morbidity of preterm infants that lacks specific therapies. Robust mechanistic preclinical studies done in the past two decades have identified several therapeutic targets and interventions that have a high translational potential to meaningfully manage infants with this disabling disease complex. Most of these are rodent studies, and their strengths include: 1) a comparable lung developmental stage and disease phenotype with preterm infants; 2) a reproducible disease phenotype with exposure to well-known insults of lung injury; 3) conducive to establishing a direct cause–effect relationship because of the feasibility of gene manipulation in these animals; 4) feasibility of obtaining a sufficient number of animals to test the hypothesis accurately; 5) timeliness with rapid turnover; 6) ease of animal husbandry; and 7) cost-effectiveness. However, there are certain disadvantages with such studies. For instance, despite the anatomic similarities between the murine lungs and preterm human lungs, there are functional differences. Murine lungs are surfactant-sufficient and do not have respiratory disease at basal conditions, whereas preterm infants are surfactant-deficient and have respiratory failure at birth. Further, the smaller size of the rodents poses technical challenges and precludes one from simulating an identical clinical scenario of a preterm infant who is at high risk of developing BPD and PH. For example, it is practically impossible to deliver positive pressure ventilation through continuous positive airway pressure (NCPAP) and mechanical ventilation or to administer intravenous parental nutrition to these small animals. Therefore, there is a need to validate these interventions and therapies in large animals such as lambs and non-human primates before designing clinical trials. The larger body and organ dimensions of these animals make it relatively easy to subject them to all the interventions of a preterm infant and accurately model human BPD and PH.

## Author Contributions

AE-S and BS participated in the conception and design, performed the literature search, analyzed and interpreted the data, drafted, revised, and approved the final version of the submitted manuscript.

## Conflict of Interest

The authors declare that the research was conducted in the absence of any commercial or financial relationships that could be construed as a potential conflict of interest.

## Abbreviations

BPD: bronchopulmonary dysplasia
BNP: brain natriuretic peptide
CTGF: connective tissue growth factor
GA: gestational age
GF: germ-free
IL: interleukin
iNO: inhaled nitric oxide
LV: left ventricle
MSCs: mesenchymal stem cells
NGF: non-germ-free
PAAT: pulmonary artery acceleration time
PH: pulmonary hypertension
PNGR: postnatal growth restriction
PVR: pulmonary vascular resistance
PVS: pulmonary vein stenosis
RV: right ventricle
SDF-1: stromal-derived factor-1
sFlt-1: soluble fms-like tyrosine kinase 1
TAPSE: tricuspid annular plane systolic excursion
TRJV: tricuspid regurgitation jet velocity
VEGF: vascular endothelial growth factor
